# Relationships between perceived health status and ambient air quality parameters in healthy Japanese: a panel study

**DOI:** 10.1186/s12889-019-6934-7

**Published:** 2019-05-22

**Authors:** Motoyuki Nakao, Keiko Yamauchi, Satoshi Mitsuma, Hisamitsu Omori, Yoko Ishihara

**Affiliations:** 1 Department of Public Health, School of Medicine, Kurume University, 67 Asahimachi, Kurume, Fukuoka, 830-0011 Japan; 2 Niigata Association of Occupational Health Inc, Niigata, Japan; 3 Department of Biomedical Laboratory Sciences, Faculty of Life Sciences, Kumamoto University, Kumamoto, Japan

**Keywords:** Air pollution, Asian sand dust, Particulate matter, Health status, COOP/WONCA chart, Respiratory symptoms, Japan

## Abstract

**Background:**

There has been growing global concern about air pollution due to its great risk to public health. In Japan, although industrial- and traffic-related air pollution has been decreasing, concerns about particulate matter air pollution has been growing in recent years. In this study, we examined the effects of air pollution on symptoms and the health status of healthy subjects in Japan.

**Methods:**

Participants (*n* = 2887) who visited healthcare centers in Kumamoto or Niigata prefectures in February from 2010 to 2015 were asked to fill out a questionnaire, which was a self-completed booklet containing questions on the characteristics of participants, their respiratory symptoms, and questionnaires on their health status in February, May, and July. Generalized estimating equation analyses were performed to predict the factors associated with the symptoms and health status using two-week averages of air quality parameters obtained from 49 monitoring stations as independent variables.

**Results:**

Only allergy was associated with air quality in both areas. Prevalence of the other respiratory symptoms were correlated with air quality only in Kumamoto. The health statuses including the ‘physical fitness’, ‘daily activities’, and ‘social activities’ domains were related only to time spent outdoors. The ‘overall health’ was associated with time spent outdoors and concentrations of nitrogen dioxide and suspended particulate matters (SPM) in Kumamoto, and with temperatures and SPM in Niigata. The ‘pain’ score was correlated with temperature and carbon monoxide concentration only in Kumamoto. In Kumamoto, the ‘quality of life (QoL)’ was worse in those who spent shorter hours outdoors, were exposed to lower humidity, higher concentrations of oxidants, SPM, and PM2.5, and who experienced more Asian sand dust (ASD) events. In Niigata, a worsened ‘QoL’ was associated with time spent outdoors, temperature, and SPM.

**Conclusions:**

The associations between air quality and the health status was found mainly in the comprehensive domain of the health status such as ‘overall health’ and ‘QoL’. The effect of short-term exposure to larger particles, such as SPM, on health status was observed when compared to smaller particles such as PM2.5 and gaseous pollutants.

## Background

Global concern about air pollution has been glowing due to its great risk to public health. According to World Health Organization (WHO), 6.5 million global deaths per year is estimated to be contributed to by air pollution. [1]. Air pollution levels have been increasing in developing countries, although those in affluent countries of the Americas, Europe, and Western Pacific have been decreasing. In developing countries in Asia, the burden of air pollution on has been disproportionately heavy with cardiovascular diseases and premature deaths [2]. During the 1950s to 1960s in Japan, also known as the high economic growth period, the major source of air pollution was sulfur dioxide produced from heavy industries, causing respiratory diseases such as asthma in the industrial area. Then, during the 1970s, air pollution due to motor vehicle exhaust containing nitrogen dioxide and particulate matters became a serious problem. This traffic-related air pollution was also linked to the onset and aggravation of childhood asthma in cities along major roads with heavy traffic [3]. These air pollutions have been decreasing through efforts by policymakers and public administrations through enforcement of legislation against air pollution such as the Basic Act for Environmental Pollution Control (1967) and the Air Pollution Control Act (1968) [4].

In recent years, air pollution in mainland East Asia is reported to be one of the most air-polluted areas in the world [5–7], and transboundary air pollution from mainland East China is the reason for renewed concern about air pollution in Japan [8, 9]. Pollutants are generated by human activities such as transportation, industries, and solid fuel combustion [10]. In addition to these anthropogenic sources, natural phenomenon called Asian sand dust (ASD, also known as “yellow sand”) also causes air pollution in Japan. ASD particles originating from Northeast Asia including the Gobi and Taklamakan deserts, and the Loess plateau travel long distance to Japan with the Westerlies and monsoon, and are accompanied by air pollutants produced from industrialized areas in the east coast of China [11–13]. Health effects of particulate matter air pollution have been reported through a perspective of mortality and morbidity of cardiopulmonary diseases, the prevalence of respiratory symptoms and hospital admissions [14–16]. Although the health effects of air pollution including mortality, symptoms, and physiological functions have been previously reported in Japan [17–20], the health-related quality of life (QoL), which refers to the multidimensional concept of a subjective perception of well-being, should also be used as an adverse health outcome [21]. In this study, we examined the effects of air pollution including ASD events on the symptoms and the health status of healthy subjects in two geographically distinct areas; Kumamoto in southwest Japan where air pollution is relatively high due to transboundary air pollution, and Niigata in the mid-north of Honshu island where air pollution is relatively mild.

## Methods

### Study design and subjects

The present studies were conducted at two healthcare centers in Japan, the Japan red cross Kumamoto healthcare center (Kumamoto prefecture) and the Niigata association of occupational health (Niigata prefecture). Participants were Japanese adults aged between 40 and 79 years, and they were recruited at the healthcare centers during their medical checkup. The questionnaires were administered to study participants in February, and the participants were followed up in May and July. The administration of the questionnaire was on site in February and by mail in May and July. The studies were started in 2010 and finished in 2015, and the participants were different each year. Subjects who agreed to participate in the present study received a self-completed questionnaire, which is the Japanese version of the questionnaire used in our previous study [22]. The questionnaire contained questions on age, gender, occupation, respiratory symptoms during last two weeks, as well as the COOP/WONCA charts (The ‘Dartmouth COOP Functional Health Assessment Charts/WONCA (World organization of Family Doctors)’). The COOP/WONCA chart is valid, reliable, and easily understandable questionnaire, and widely used to measure multidimensional health status including health-related quality of life (QoL) during last two weeks. For the Japanese version of the COOP/WONCA charts, permission was obtained from the Japan Primary Care Association. The COOP/WONCA charts consists of eight items (physical fitness; feelings; daily activities; social activities; change in health; overall health; pain; QoL) [23]. Responses to these items were scored on a five-point ordinal scale ranging from 1 to 5 (1 = best, 5 = worst). At the time of administration of the first questionnaire, physician conducted body measurements, medical interviews, auscultation, and some examination as part of the medical checkup. Subjects diagnosed as severe diseases or having history of severe diseases such as cancer or pneumonectomy, who did not complete questionnaires, or did not undergo proper checkup were excluded from further analysis.

### Air quality data

**Fig. 1:**
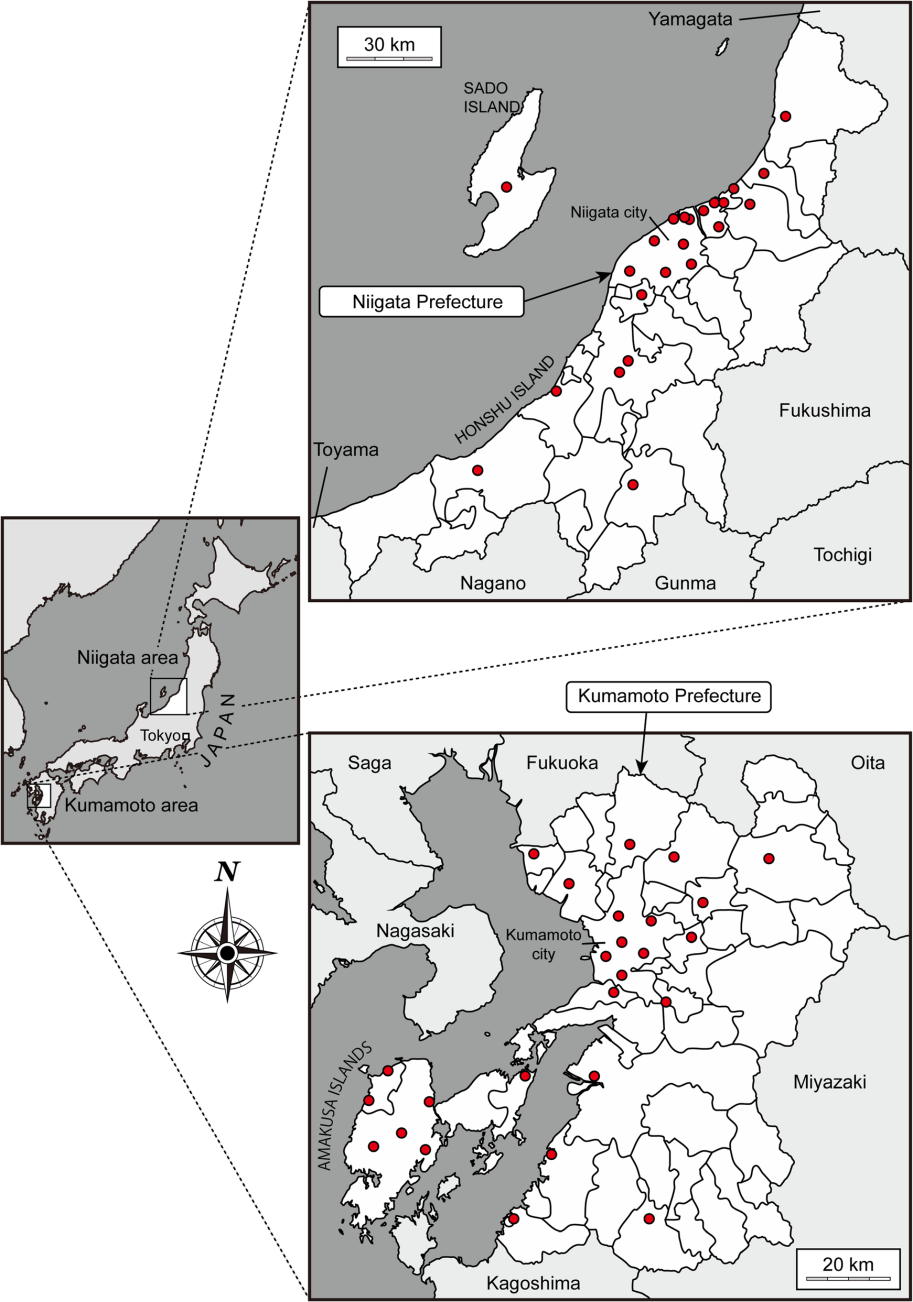
Study area and the location of monitoring stations. Left panel shows map of Japan. Niigata and Kumamoto prefecture were shown in the right-upper panel and right-lower panel, respectively. Monitoring stations were presented as red circles. The original data of the map was obtained from http://d-maps.com/index.php?lang=en

Air monitoring data were obtained from local public administration (Kumamoto and Niigata prefectures) and the Ministry of the Environment of Japan. Measuring method of the data including carbon monoxide, nitrogen dioxide, photochemical oxidants (oxidants), sulfur dioxide, particulate matter less than 2.5 μm in diameter (PM2.5), suspended particulate matters (SPM) were according to the ministry of the environment of Japan [24]. SPM was defined as airborne particles with a diameter ≤ 10 μm and oxidants were oxidizing substances such as ozone and peroxyacetyl nitrate produced by photochemical reactions (only those capable of isolating iodine from neutral potassium iodide, excluding nitrogen dioxide) [24]. Data obtained at the nearest monitoring station of the residential addresses of each participant was considered as the subject’s environmental exposure. The map of the survey area was shown in Fig. 1. The number of monitoring stations used for exposure assessment were 26 in Kumamoto and 23 in Niigata. The distance from each participants’ address were shown in Table 1.

**Table 1:** Characteristics of participants

	February				May				July			
	Kumamoto		Niigata		Kumamoto		Niigata		Kumamoto		Niigata	
N	1537		1350		1101		1119		905		987	
	100%		100%		71.6% v. Feb		82.9% v. Feb		58.9% v. Feb		73.1% v. Feb	
Year of survey	N		N		N	v. Feb	N	v. Feb	N	v. Feb	N	v. Feb
2010	186	12.1%	–	–	121	65.1%	–	–	104	55.9%	–	–
2011	212	13.8%	198	14.7%	159	75.0%	152	76.8%	140	66.0%	132	66.7%
2012	306	19.9%	299	22.1%	234	76.5%	225	75.3%	196	64.1%	192	64.2%
2013	287	18.7%	243	18.0%	197	68.6%	212	87.2%	163	56.8%	191	78.6%
2014	274	17.8%	305	22.6%	202	73.7%	266	87.2%	155	56.6%	230	75.4%
2015	272	17.7%	305	22.6%	188	69.1%	264	86.6%	147	54.0%	242	79.3%
Age												
years old (mean ± SD)	64.4 ± 9.8		59.1 ± 6.2		65.9 ± 9.5		59.5 ± 6.1		66.7 ± 9.5		59.7 ± 6.1	
Gender	N		N		N		N		N		N	
Male	1089	70.9%	939	69.6%	798	72.5%	771	68.9%	663	73.3%	674	68.3%
Female	448	29.2%	411	30.4%	303	27.5%	348	31.1%	242	26.7%	313	31.7%
Body mass index												
kg/m^2^ (mean ± SD)	23.6 ± 3.1		23.2 ± 3.1		23.4 ± 2.9		23.1 ± 3.0		23.3 ± 2.9		23.0 ± 3.0	
Smoking status	N		N		N		N		N		N	
Current smoker	210	13.7%	312	23.1%	121	11.0%	232	20.7%	89	9.8%	192	19.5%
Former smoker	561	36.5%	481	35.6%	430	39.1%	422	37.7%	364	40.2%	367	37.2%
Never smoker	766	49.8%	557	41.3%	550	50.0%	465	41.6%	452	49.9%	428	43.4%
Working status	N		N		N		N		N		N	
Active worker	951	63.6%	1169	86.9%	608	56.1%	939	84.1%	463	52.0%	820	83.3%
Retired/ Not in employment	544	36.4%	177	13.2%	476	43.9%	178	15.9%	428	48.0%	165	16.8%
Time spent outdoors												
Hours	4.0 ± 3.2		4.7 ± 4.2		4.2 ± 3.2		4.1 ± 3.6		4.0 ± 3.1		3.9 ± 3.4	
Prevalence of respiratory symptoms												
1. Weather affects cough	88	5.8%	86	6.4%	76	7.1%	66	6.0%	37	4.2%	59	6.1%
2. Sputum production without a cold	391	25.5%	397	29.5%	303	28.4%	320	28.9%	222	25.0%	253	26.0%
3. Sputum production in early morning	258	16.9%	213	15.8%	193	18.1%	172	15.6%	142	16.0%	160	16.6%
4. Frequent wheezes	123	8.0%	108	8.0%	89	8.3%	91	8.3%	54	6.1%	70	7.2%
5. Allergy	307	20.0%	232	17.3%	239	22.1%	239	21.6%	145	16.3%	164	16.9%
Monitoring station												
Number of stations	26		23									
Distance from each residence												
Median (Min. – Max.) (km)	3.70 (0.06–33.43)		3.31 (0.10–30.88)									

### Data handling and statistical analyses

Data obtained in this study were anonymized and administered as electronic data for the analysis. To make the scores of the COOP/WONCA charts available for generalized estimating equation (GEE) analyses, we dichotomized the COOP/WONCA chart scores as dependent variables (0 for better scores (1, 2 points for the ‘physical fitness’ and ‘feelings’; 1–3 points for the ‘daily activities’, ‘social activities’, ‘change in health’, ‘overall health’, ‘pain’, and ‘QoL’); 1 for worse scores (3–5 points for the ‘physical fitness’ and ‘feelings’; 4, 5 points for the ‘daily activities’, ‘social activities’, ‘change in health’, ‘overall health’, ‘pain’, and ‘QoL’)). Respiratory symptoms (Q1, Does the weather affect your cough?; Q2, Have you ever coughed up sputum from your chest when you do not have a cold?; Q3, Do you usually cough up sputum from your chest first thing in the morning?; Q4, How frequently do you wheeze?; Q5 Do you have or have you had any allergies? (0, No (Q1–3 and 5) or Never (Q4); 1, Yes (Q1–3 and 5) or Occasionally or more often (Q4))) were also employed as dependent variables for GEE analysis. All odds ratios (OR) were adjusted by year of survey, age, gender, body mass index (BMI), smoking status, working status, and subsequently, parameters representing environmental exposure including time spent outdoors, ambient temperature, relative humidity, carbon monoxide, nitrogen dioxide, oxidants, sulfur dioxide, PM2.5, SPM, or number of ASD events were entered separately (see *Independent variables*). Statistical analyses were performed using the statistical software package SPSS ver. 22 (IBM Corporation, Armonk, NY, USA). *P* values of less than 0.05 were considered statistically significant.

### Independent variables

For GEE analysis, ORs were adjusted by the independent variables including year of survey (2010–2015), age (years old), gender (0, female; 1, male), BMI (kg/m^2^), current smoking status (0, non-smoker; 1 ex-smoker; 2, current smoker), and working status (0, currently working; 1, retired or not in employment). For the parameters regarding exposure to ambient air, the ORs were presented as incremental difference of one hour spent outdoors, 10 °C of ambient temperature, 10% of relative humidity, one ASD event, 10 ppb of carbon monoxide, nitrogen dioxide, oxidants, and sulfur dioxide, and 10 μg/m^3^ of PM2.5 and SPM. For carbon monoxide, nitrogen dioxide, sulfur dioxide, PM2.5, and SPM, the mean of the preceding 14 daily mean values was entered as the independent variable. The mean of the preceding 14 mean values from 6 am to 8 pm of each day was entered as the independent variable of oxidants. For ASD events, the number of ASD events in the month prior to each survey was entered as the independent variable. All independent variables except for air quality data were included in each model. Air quality variables were entered separately because these air quality parameters vary in relation to each other.

## Results

### Participant characteristics

Table 1 shows the participants’ characteristics. During the month of February from 2010 to 2015, a total of 1537 and 1350 subjects in Kumamoto and Niigata prefectures, respectively, participated in this study. The overall follow-up rate was higher in Niigata prefecture than that in Kumamoto prefecture. Male participants were more than twice as much as female participants in both regions. The percentage of active worker was higher in Niigata prefecture than that in Kumamoto prefecture, reflecting the younger mean age of participants in Niigata prefecture when compared to that in Kumamoto prefecture. Current smoking rate in Niigata prefecture was nearly twice of that in Kumamoto prefecture. The prevalence of symptoms were similar between Kumamoto and Niigata prefecture.

### Seasonal variations of ambient air quality during the present study

**Fig. 2:**
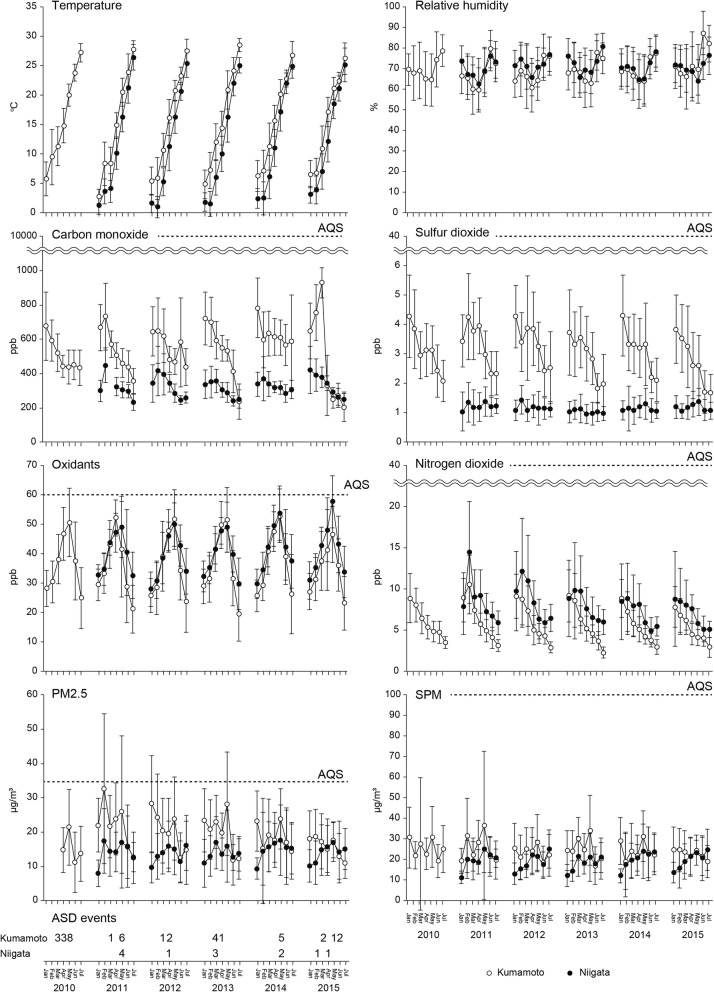
Temporal variations of air quality parameters during January 2010 – July 2015. The monthly averages of the daily mean values of temperature, relative humidity, the concentrations of carbon monoxide, nitrogen dioxide, oxidants, sulfur dioxide, PM2.5, and SPM, and the number of ASD events per month. Data were collected from the ministry of environment of Japan and all the monitoring stations from which data were available. Data except for the number of ASD events were presented as mean ± SD. Open circles and closed circles represent the data in Kumamoto and Niigata, respectively. The national ambient AQS is shown as a dotted line

The monthly mean of all available air quality data during January to July is presented in Fig. 2. The temperature in Kumamoto was generally higher than in Niigata. Relative humidity was relatively higher in June and July during the rainy season in both areas. Carbon monoxide and sulfur dioxide concentrations were higher in Kumamoto than in Niigata, and the concentrations were higher in the winter months than in the spring and summer. Additionally, these concentrations were well below air quality standard (AQS) in Japan. Oxidant concentration in Kumamoto was comparable with that of Niigata, and these levels tended to be high during March to May in both areas. Although the monthly average of the daily mean oxidant levels did not reach AQS, there were many days that the hourly oxidant level during daytime (from 6 am to 8 pm) exceeded AQS. Nitrogen dioxide concentrations tended to be higher in Niigata compared to Kumamoto, and was high in the winter months and gradually decreased in the following months. Additionally, these concentrations were well below AQS in Japan. Monthly mean PM2.5 levels were relatively higher in Kumamoto than in Niigata, although there were large fluctuations in the intra-month levels in both areas. This fluctuation was also observed for SPM levels. There were several days that the daily PM2.5 and SPM levels exceeded AQS while the monthly means of both were below AQS. There were more ASD events in Kumamoto than in Niigata every year and almost all events occurred during springtime. The total number of ASD events was 39 in Kumamoto during 2010–2015 and 12 in Niigata during 2011–2015.

### Association of ambient air pollutant exposure with subjective respiratory symptoms

GEE analysis was used to identify the association between variables regarding air pollution and respiratory symptoms (Table 2). In Kumamoto prefecture, symptom 1 (weather affects a cough) was positively associated with the concentrations of oxidants, SPM, and PM2.5, and the number of ASD events. Symptom 2 (sputum production without a cold) was increased in association with the concentrations of oxidants, SPM, and PM2.5. Symptoms 1, 3 (sputum production first thing in the morning), 4 (frequent wheezes), and 5 (allergy) were negatively associated with an increment in relative humidity and positively associated with oxidants concentration. Additionally, symptom 5 was positively correlated with SPM and PM2.5 concentrations. In Niigata, only symptom 5 was significantly associated with a shorter time spent outdoors, lower relative humidity, higher concentrations of carbon monoxide, oxidants, and SPM, and ASD events.

**Table 2:** Associations between variables regarding ambient air pollutant exposure and respiratory symptoms

	Dependent variables (0, no symptom; 1, having symptom)									
	Symptom 1		Symptom 2		Symptom 3		Symptom 4		Symptom 5	
Independent variables	OR	95% CI	OR	95% CI	OR	95% CI	OR	95% CI	OR	95% CI
Kumamoto										
Hours spent outdoors (each 1 h)	1.01	0.96–1.07	0.97	0.94–1.01	0.97	0.93–101	1.01	0.97–1.07	0.99	0.95–1.03
Temperature (each 10 °C)	0.93	0.79–1.09	1.05	0.97–1.14	0.98	0.89–1.07	0.92	0.79–1.06	0.95	0.88–1.04
Relative humidity (each 10%)	0.78	0.67–0.90	0.95	0.89–1.02	0.94	0.73–0.96	0.84	0.73–0.96	0.86	0.79–0.93
Carbon monoxide (each 10 ppb)	1.00	0.99–1.01	1.00	1.00–1.00	1.00	1.00–1.01	1.00	1.00–1.01	1.00	1.00–1.01
Nitrogen dioxide (each 10 ppb)	1.08	0.83–1.44	1.01	0.87–1.17	1.15	0.97–1.37	1.18	0.93–1.49	1.12	0.94–1.31
Oxidants (each 10 ppb)	1.16	1.06–1.27	1.06	1.01–1.12	1.03	0.98–1.09	1.10	1.02–1.20	1.10	1.04–1.16
Sulfur dioxide (each 10 ppb)	0.65	0.21–2.00	0.34	0.17–0.67	0.57	0.24–1.33	0.61	0.16–2.22	1.45	0.71–2.97
SPM (each 10 μg/m^3^)	1.17	1.02–1.34	1.10	1.02–1.20	1.08	0.99–1.18	1.07	0.95–1.21	1.15	1.06–1.26
PM2.5 (each 10 μg/m^3^)	1.17	1.03–1.33	1.17	1.03–1.33	1.10	0.96–1.27	1.18	0.94–1.49	1.32	1.15–1.52
Asian sand dust event (each 1 event in last one month)	1.11	1.05–1.17	1.03	1.00–1.05	0.99	0.96–1.02	1.03	0.98–1.09	1.03	1.00–1.07
Niigata										
Hours spent outdoors (each 1 h)	1.01	0.97–1.06	1.00	0.97–1.03	0.99	0.96–1.03	1.02	0.98–1.06	0.96	0.93–0.99
Temperature (each 10 °C)	0.98	0.84–1.14	0.93	0.87–1.00	1.04	0.95–1.14	0.98	0.85–1.12	1.02	0.94–1.09
Relative humidity (each 10%)	1.08	0.83–1.42	0.90	0.79–1.03	1.13	0.97–1.32	0.97	0.74–1.27	0.71	0.61–0.83
Carbon monoxide (each 10 ppb)	1.01	0.98–1.04	1.00	0.98–1.02	0.99	0.97–1.01	0.99	0.97–1.02	1.02	1.00–1.04
Nitrogen dioxide (each 10 ppb)	0.73	0.46–1.17	0.93	0.72–1.21	0.96	0.69–1.32	0.83	0.55–1.22	1.03	0.78–1.38
Oxidants (each 10 ppb)	0.96	0.87–1.06	1.02	0.97–1.07	0.98	0.92–1.04	1.03	0.94–1.14	1.14	1.07–1.21
Sulfur dioxide (each 10 ppb)	1.93	0.09–45.52	0.95	0.15–5.89	0.51	0.05–5.69	1.02	0.05–21.65	2.26	0.30–17.19
SPM (each 10 μg/m^3^)	0.96	0.78–1.18	0.93	0.83–1.04	0.97	0.84–1.12	0.93	0.77–1.12	1.16	1.02–1.32
PM2.5 (each 10 μg/m^3^)	0.90	0.63–1.31	0.94	0.77–1.15	0.94	0.72–1.23	0.95	0.66–1.36	1.20	0.96–1.49
Asian sand dust event (each 1 event in last one month)	0.98	0.87–1.11	0.98	0.92–1.04	1.01	0.93–1.09	1.08	0.97–1.19	1.10	1.03–1.18

Symptom 1, weather affects a cough; symptom 2, coughing up sputum when the subject does not have a cold; symptom 3, coughing up sputum first thing in the morning; symptom 4, frequent wheeze; symptom 5, allergic symptoms. Odds ratios (OR) were adjusted by year of survey, gender, age, body mass index (kg/m^2^), smoking status (current, former, or never smoker), working status (active worker or retired/ not in employment). CI, confidence interval; ppb, part per billion; SPM, Suspended particulate matter; PM2.5, Particulate matter less than 2.5 μm in diameter

### Association of ambient air pollutant exposure with health status measured by the COOP/WONCA chart

GEE analysis was also used to identify the association between variables regarding air pollution and the health status measured by the COOP/WONCA chart (Table 3). Shorter hours spent outdoors was associated with worse scores for ‘physical fitness’, ‘daily activities’, ‘social activities’, ‘overall health’, and ‘QoL’ items in Kumamoto, and for ‘physical fitness’, ‘social activities’, and ‘quality of life’ items in Niigata. Regarding ambient temperature, only the ‘pain’ item was negatively associated in Kumamoto, while it was positively associated with ‘overall health’ and ‘QoL’ items in Niigata. In Kumamoto, relative humidity was negatively correlated only with the ‘QoL’ item, higher carbon monoxide concentration was only associated with a worse score for the ‘pain’ item, higher nitrogen dioxide and SPM concentrations were associated with the ‘overall health’ item, and SPM also associated with a worse score for the ‘QoL’ item. The ‘QoL’ item was also associated with PM2.5 concentration and number of ASD events in Kumamoto. In Niigata, higher SPM concentration was associated with a worse score for ‘overall health’ and ‘QoL’ items.

**Table 3:** Associations between variables regarding ambient air pollutant exposure and health status measured by the COOP/WONCA chart

	Dependent variables (COOP/ WONCA chart items) (0, better; 1, worse)															
	Physical fitness		Feelings		Daily activities		Social activities		Change in health		Overall health		Pain		Quality of life	
Independent variables	OR	95% CI	OR	95% CI	OR	95% CI	OR	95% CI	OR	95% CI	OR	95% CI	OR	95% CI	OR	95% CI
Kumamoto																
Hours spent outdoors (each 1 h)	0.94	0.92–0.97	0.98	0.95–1.01	0.81	0.72–0.91	0.74	0.63–0.86	0.99	0.93–1.05	0.93	0.88–0.99	0.99	0.92–1.06	0.92	0.87–0.98
Temperature (each 10 °C)	0.93	0.85–1.03	0.97	0.87–1.08	1.06	0.78–1.42	0.95	0.62–1.44	1.01	0.80–1.27	0.87	0.73–1.03	0.82	0.69–0.97	1.02	0.85–1.24
Relative humidity (each 10%)	1.06	0.98–1.16	0.97	0.88–1.07	1.00	0.76–1.31	0.93	0.64–1.36	0.99	0.79–1.23	0.94	0.80–1.12	1.03	0.90–1.21	0.80	0.66–0.96
Carbon monoxide (each 10 ppb)	1.00	1.00–1.01	1.00	1.00–1.01	1.00	0.98–1.01	1.01	0.99–1.02	1.00	0.99–1.01	1.01	1.00–1.01	1.01	1.00–1.01	1.00	1.00–1.01
Nitrogen dioxide (each 10 ppb)	1.13	0.98–1.31	1.03	0.88–1.21	0.83	0.57–1.22	1.12	0.70–1.77	1.16	0.88–1.54	1.36	1.08–1.69	1.21	0.93–1.57	1.26	0.98–1.63
Oxidants (each 10 ppb)	0.97	0.92–1.01	1.01	0.96–1.06	1.01	0.87–1.17	1.08	0.91–1.28	1.05	0.94–1.17	1.03	0.95–1.12	0.99	0.92–1.07	1.13	1.03–1.23
Sulfur dioxide (each 10 ppb)	1.09	0.61–1.95	1.27	0.67–2.41	1.71	0.36–7.93	2.30	0.28–18.67	0.90	0.25–3.28	0.85	0.30–2.41	1.07	0.36–3.19	0.75	0.21–2.62
SPM (each 10 μg/m^3^)	1.06	0.99–1.14	1.03	0.94–1.12	0.91	0.75–1.10	0.99	0.78–1.24	1.15	0.98–1.34	1.13	1.00–1.28	0.99	0.86–1.15	1.22	1.07–1.38
PM2.5 (each 10 μg/m^3^)	1.00	0.89–1.13	1.06	0.92–1.22	0.93	0.67–1.29	1.28	0.82–2.02	1.06	0.82–1.38	1.13	0.92–1.38	1.12	0.92–1.36	1.27	1.02–1.58
Asian sand dust event (each 1 event in last one month)	0.99	0.97–1.02	1.00	0.96–1.03	0.96	0.86–1.08	0.91	0.78–1.05	1.06	0.99–1.13	1.03	0.98–1.08	0.98	0.93–1.03	1.10	1.03–1.17
Niigata																
Hours spent outdoors (each 1 h)	0.96	0.93–0.98	0.98	0.96–1.01	0.94	0.88–1.00	0.91	0.84–0.99	0.98	0.94–1.02	0.99	0.95–1.03	1.01	0.97–1.05	0.95	0.91–1.00
Temperature (each 10 °C)	1.01	0.94–1.09	0.94	0.87–1.01	1.02	0.83–1.26	1.00	0.74–1.34	1.18	0.98–1.41	1.17	1.02–1.34	0.93	0.83–1.06	1.20	1.03–1.38
Relative humidity (each 10%)	1.09	0.95–1.27	0.93	0.80–1.09	0.90	0.61–1.32	1.44	0.73–2.84	0.84	0.59–1.21	0.99	0.69–1.16	1.08	0.83–1.44	0.80	0.60–1.07
Carbon monoxide (each 10 ppb)	1.00	0.99–1.02	1.00	0.99–1.02	1.02	0.97–1.07	1.02	0.95–1.08	0.99	0.96–1.03	1.00	0.97–1.02	1.00	0.98–1.03	1.00	0.97–1.04
Nitrogen dioxide (each 10 ppb)	0.96	0.77–1.20	0.94	0.76–1.17	1.22	0.75–1.97	0.77	0.34–1.74	0.93	0.62–1.40	0.90	0.63–1.28	0.99	0.70–1.40	1.02	0.71–1.49
Oxidants (each 10 ppb)	0.98	0.93–1.03	1.03	0.97–1.08	1.04	0.90–1.20	0.88	0.70–1.10	1.06	0.93–1.20	1.06	0.97–1.16	0.98	0.90–1.08	1.05	0.95–1.17
Sulfur dioxide (each 10 ppb)	0.72	0.12–4.12	0.33	0.07–1.61	0.09	0.00–7.36	0.13	0.00–67.14	1.26	0.04–42.23	2.59	0.16–41.08	0.33	0.01–20.71	0.60	0.03–10.49
SPM (each 10 μg/m^3^)	1.05	0.94–1.16	0.91	0.82–1.01	1.16	0.89–1.52	1.37	0.95–1.99	1.22	0.96–1.54	1.20	1.01–1.40	0.99	0.84–1.17	1.24	1.04–1.49
PM2.5 (each 10 μg/m^3^)	1.03	0.85–1.23	0.97	0.80–1.17	0.78	0.48–1.27	0.86	0.41–1.81	1.09	0.71–1.68	1.32	0.97–1.79	0.97	0.71–1.34	1.36	0.98–1.89
Asian sand dust event (each 1 event in last one month)	0.98	0.92–1.04	0.98	0.92–1.05	0.91	0.75–1.10	1.06	0.85–1.33	1.02	0.89–1.18	0.95	0.84–1.06	1.01	0.91–1.12	0.98	0.86–1.11

ORs were adjusted by year of survey, gender, age, body mass index (kg/m^2^), smoking status (current, former, or never smoker), working status (active worker or retired/ not in employment)

CI, confidence interval; ppb, part per billion; SPM, Suspended particulate matter; PM2.5, Particulate matter less than 2.5 μm in diameter

## Discussion

We carried out a health survey on 2887 Japanese middle-aged or elderly subjects who visited a healthcare center for a medical checkup in Kumamoto and Niigata prefectures during 2010–2015, and the participants were different for each year. The survey was a three time-point questionnaire survey. The first survey was held in February when air pollution is relatively severe. The participants were followed up in May when ASD events have been frequently observed in the Western part of Japan. The last survey was held in July when the ambient air is the cleanest. A GEE approach was applied to identify the environmental factors associated with subjects’ respiratory symptoms and the health status as measured by the COOP/WONCA chart. The association of air quality parameters with respiratory symptoms and several aspects of the health status were found in both areas.

Health effects of ambient air pollution have been reported several decades ago. In particular, the Great Smog of London of December 1952 is a well-known episode of acute exposure to lethal smog [25]. Air pollutants such as sulfur dioxide derived from coal-burning homes, coal-fired power plants, and factories caused heavy smog under the condition of temperature inversion due to windless cold weather [26]. This episode resulted in thousands of excessive death and sickness. Since then, numerous studies regarding the effect of air pollution on health such as cardiopulmonary mortality and morbidity has been published [16, 27]. In the present study, in Kumamoto, increased concentrations of oxidants, SPM, and PM2.5 were associated with symptoms 1 (weather affects a cough), 2 (sputum production without a cold), and 5 (allergy) (Table 2). Symptoms 1 and 5 were also related to the number of ASD events and lower relative humidity, respectively. Symptom 3 (sputum production first thing in the morning) was negatively associated with relative humidity. Symptom 4 (frequent wheezes) was increased in association with higher oxidant concentration and lower humidity. In contrast to Kumamoto, only symptom 5 was associated with parameters regarding ambient air pollution exposure in Niigata. Symptom 5 (Allergy) was the most sensitive to humidity levels and air pollutants in both areas. In some cases of environmental exposure, allergic or asthma-like symptoms has been reported to occur without clinical signs [28]. It is, therefore, unsurprising that the healthy subjects in this study presented with these respiratory symptoms. The monthly averages of the daily mean ambient temperature, relative humidity, and air pollutant concentrations were calculated and are shown in Fig. 2, and the air pollutant concentrations were generally lower in Niigata than those in Kumamoto with a few exceptions. It is suggested to be the reason that the associations between air pollution and respiratory symptoms were weaker in Niigata than in Kumamoto. In the present study, presentation of allergic symptoms might have a lower threshold for environment exposure compared with other symptoms as it was the symptom most widely associated with the environment in both regions. Oxidant concentration was significantly associated with four kinds of symptoms in Kumamoto. Adverse health effects of ozone ranging from respiratory symptoms to increased mortality have been well-documented [29, 30]. Exposure to ozone has been reported to enhance airway hyperreactivity at frequently observed concentrations even in healthy subjects [31, 32]. The result of the present study was consistent with these previous studies. Particulate matters were associated with cough, sputum, and allergic symptoms in Kumamoto, and to allergic symptom in Niigata (Table 2). Health effects of acute exposure to particulate matter air pollution have widely been evaluated using end points such as death, cardiovascular mortality, hospitalization, healthcare visit, lung function, and respiratory symptoms [14–16, 33]. In addition to the anthropogenic origin of air pollution, ASD events also have a great influence on the particulate matter concentration in East Asia including Japan [11–13]. ASD events were reported to be associated with increased cardiopulmonary mortality and morbidity in Japan [34–39]. An increased number of ASD events was significantly associated with symptom 1 in Kumamoto and symptom 5 in Niigata (Table 2). In Kumamoto, the number of ASD events also showed positive association with Symptoms 2 and 5 with ORs of 1.03 (95% confidence interval (CI): 1.00–1.05, not significant) and 1.03 (1.00–1.07, not significant), respectively. Most existing reports regarding the health effects of air pollution have been focused on children or patients with cardiopulmonary diseases because adverse effects were likely to be more obvious in these subjects. All the subjects who participated in the present study were adults and did not suffer from any chronic cardiorespiratory diseases. These results suggest that there are apparent associations between air pollution and respiratory symptoms even in healthy adult subjects.

According to the ATS statement, the concept of health-related QoL, which refers to the individual’s perception of well-being, should also be considered as an adverse health outcome of air pollution together with mortality, clinical outcomes, physiological impact, biomarkers, and symptoms [21]. In the present study, we administered the COOP/WONCA chart to measure the health status including the QoL domain. Subjects who spent shorter hours outdoors were associated with decreased scores of ‘physical fitness’, ‘social activities’, and ‘QoL’ domains both in Kumamoto and Niigata (Table 3). This result suggests that those who spent longer hours outdoors lived more fulfilling lives than those who were more likely to stay indoors. A lower temperature was related to the worse score for ‘pain’ in Kumamoto while worse scores for ‘overall health’ and ‘QoL’ were decreased in association with higher temperature in Niigata (Table 3). Humidity was associated with the ‘QoL’ score only in Kumamoto. Both higher and lower ambient temperatures were reported to have adverse effects on the risk of cardiovascular diseases [40, 41]. For physical activity, a study in Japan showed that the daily step count peaks at the ambient temperature of 17 °C, while physical activity decreased at higher or lower temperatures [42]. In contrast, Giorgini et al., reported that a higher temperature was associated with a decrease in aerobic exercise capacity, leading to a worse QoL in patients undergoing cardiac rehabilitation [43]. The result in Niigata from the present study was consistent with this report, suggesting that higher ambient temperature is associated with a detrimental change in the ‘overall health’, and ‘QoL’ even in healthy subjects. In parallel, the low temperature was reported to have a significant impact on subjective pain score in a remarkable proportion of the population [44, 45], which is also consistent with our result in Kumamoto. Regarding meteorological conditions, further research is needed to verify the relationships between the health status and the climate parameters including rainfall, barometric variation, and abrupt weather changes. Although of a weaker association, higher carbon monoxide concentration was significantly associated with a worse score for the ‘pain’ scale in Kumamoto (Table 3). Higher nitrogen dioxide concentration was related to a decreased ‘overall health’ score, and higher PM2.5 concentration and increased number of ASD events were associated with a worse score for the ‘QoL’ in Kumamoto. Increased concentration of SPM was associated with worse scores for the ‘overall health’ and ‘QoL’ in both areas. Two Japanese studies showed that the concentrations of nitrogen dioxide and oxidants were significantly associated with the ‘vitality’ domain of the Medical Outcome Study Short Form-36 Health Survey (SF-36) [46, 47]. The ‘vitality’ domain is a constituent of the mental component score of the SF-36, while the corresponding mental component in the present study is the “feelings” scale. In contrast to the previous studies, exposure to air pollution in healthy subjects in this study was not significantly associated with the ‘feelings’ scale, but was instead associated predominantly with comprehensive domains such as ‘overall health’ and ‘QoL’. This difference between studies might be attributed to differences in the study population, study design, questionnaire, or analytical method. These results suggested that further studies with a consistent design is needed to clarify the exact effects of air pollution and their magnitude. The relationships between the environmental exposure and the health status were area dependent (Table 3). Concentrations of carbon monoxide and nitrogen dioxide were both higher in Kumamoto than in Niigata (Fig. 2), suggesting that high concentrations of these pollutants resulted in significant associations with ‘pain’ or ‘overall health’ only in Kumamoto. The associations of ‘QoL’ with PM2.5 concentration and the number of ASD events were significant only in Kumamoto (Table 3). High concentration of PM2.5 in Kumamoto was suggested to be transboundary air pollution from mainland Asia [8, 9] because Kumamoto is located in Western Japan, leeward of Westerlies. For the same reason, ASD events were observed more frequently in Kumamoto than in Niigata (Fig. 2). The frequent air pollution episodes and higher pollutant concentrations were suggested to lead to a significant association between the health status and pollution only in Kumamoto. SPM concentration showed significant relationships with ‘overall health’ and ‘QoL’ items in Kumamoto and Niigata (Table 3). This result suggests that individuals exposed to SPM might be prone to perceived decreased health status although the monthly average of the daily mean concentration of SPM was well below the AQS in both areas (Fig. 2).

There are several limitations in this study. First, due to the study design as a panel study, the participants of the present study were not strictly followed. Although more information was obtained when compared with a cross-sectional study, the causal relationship between health status and parameters regarding exposure to ambient air, remains unclear. Second, we adjusted some sociodemographic factors such as age, gender, and working status to estimate the association of environmental exposure with the symptom and health status. As we carried out the present study in two distant locations of which subject and geographical characteristics were very different, the possibilities of the existence of the unknown unadjusted confounders cannot be excluded. Third, although air quality data were obtained from the monitoring stations which were located at the nearest address of each participant, it was impossible to verify the consistency between the range of movement of each participant and the location of the monitoring station. To estimate the accurate relationship between health status and ambient air pollution exposure, it is necessary to measure indoor air quality and personal exposure to air pollution. To the respect of indoor air quality, environmental tobacco smoke or secondhand smoking at home might be used as a proxy for indoor air pollution although the information about passive smoking was not collected in the present study.

## Conclusions

The associations between air quality and perceived health status were found mainly in the comprehensive domain of the health status such as “overall health” and “QoL”. From the results of the present study, we speculate that the significant effects of short-term exposure to relatively large particulate matter such as SPM on health status was more readily observed when compared with the smaller particles such as PM2.5 and gaseous pollutants.

## Abbreviations

AQS: Air quality standard
ASD: Asian sand dust
BMI: Body mass index
CI: Confidence interval
GEE: Generalized estimating equation
OR: Odds ratio
PM2.5: Particulate matters less than 2.5 μm in a diameter
QoL: Quality of life
SD: Standard deviation
SPM: Suspended particulate matters
WHO: World Health Organization
